# Artificial intelligence-based epigenomic, transcriptomic and histologic signatures of tobacco use in oral squamous cell carcinoma

**DOI:** 10.1038/s41698-024-00605-x

**Published:** 2024-06-08

**Authors:** Chi T. Viet, Kesava R. Asam, Gary Yu, Emma C. Dyer, Sara Kochanny, Carissa M. Thomas, Nicholas F. Callahan, Anthony B. Morlandt, Allen C. Cheng, Ashish A. Patel, Dylan F. Roden, Simon Young, James Melville, Jonathan Shum, Paul C. Walker, Khanh K. Nguyen, Stephanie N. Kidd, Steve C. Lee, Gretchen S. Folk, Dan T. Viet, Anupama Grandhi, Jeremy Deisch, Yi Ye, Fatemeh Momen-Heravi, Alexander T. Pearson, Bradley E. Aouizerat

**Affiliations:** 1 Department of Oral and Maxillofacial Surgery, Loma Linda University School of Dentistry, Loma Linda, CA USA; 2 Department of Oral and Maxillofacial Surgery, New York University College of Dentistry, New York, NY USA; 3 Translational Research Center, New York University College of Dentistry, New York, NY USA; 4 New York University Rory Meyers College of Nursing, New York, NY USA; 5 Department of Medicine, Section of Hematology/Oncology, University of Chicago Medical Center, Chicago, IL USA; 6 Department of Otolaryngology, University of Alabama at Birmingham, Birmingham, AL USA; 7 Department of Oral and Maxillofacial Surgery, University of Illinois Chicago, College of Dentistry, Chicago, IL USA; 8 Department of Oral and Maxillofacial Surgery, University of Alabama at Birmingham, Birmingham, AL USA; 9 Head and Neck Surgery, Providence Cancer Institute, Portland, OR USA; 10 Head and Neck Surgery, Legacy Cancer Center, Portland, OR USA; 11 Department of Otolaryngology, Rutgers New Jersey Medical School, Newark, NJ USA; 12 Katz Department of Oral & Maxillofacial Surgery, The University of Texas Health Science Center at Houston, School of Dentistry, Houston, TX USA; 13 Department of Otolaryngology, Loma Linda University School of Medicine, Loma Linda, CA USA; 14 Scripps Oral Pathology, San Diego, CA USA; 15 Rocky Vista University, Ivins, UT USA; 16 Department of Pathology and Human Anatomy, Loma Linda University School of Medicine, Loma Linda, CA USA; 17 Section of Oral, Diagnostic and Rehabilitation Sciences, College of Dental Medicine, Columbia University, New York, NY USA; 18 Herbert Irving Comprehensive Cancer Center, Columbia University Medical Center, New York, NY USA

**Keywords:** Oral cancer, Computational biology and bioinformatics, Cancer genomics

## Abstract

Oral squamous cell carcinoma (OSCC) biomarker studies rarely employ multi-omic biomarker strategies and pertinent clinicopathologic characteristics to predict mortality. In this study we determine for the first time a combined epigenetic, gene expression, and histology signature that differentiates between patients with different tobacco use history (heavy tobacco use with ≥10 pack years vs. no tobacco use). Using The Cancer Genome Atlas (TCGA) cohort (*n* = 257) and an internal cohort (*n* = 40), we identify 3 epigenetic markers (GPR15, GNG12, GDNF) and 13 expression markers (IGHA2, SCG5, RPL3L, NTRK1, CD96, BMP6, TFPI2, EFEMP2, RYR3, DMTN, GPD2, BAALC, and FMO3), which are dysregulated in OSCC patients who were never smokers vs. those who have a ≥ 10 pack year history. While mortality risk prediction based on smoking status and clinicopathologic covariates alone is inaccurate (c-statistic = 0.57), the combined epigenetic/expression and histologic signature has a c-statistic = 0.9409 in predicting 5-year mortality in OSCC patients.

## Introduction

Oral squamous cell carcinoma (OSCC) is on the rise^1^, with 30,000 new diagnoses each year in the United State alone. Five-year survival rate is as low as 60% even for early stage cancer patients. This low survival rate is in contrast to other cancers, or even other head and neck cancer subtypes, such as oropharyngeal SCC, which have significantly improved survival, due to accurate risk assessment using biomarkers and development of targeted therapeutics. Currently, clinicopathologic factors are used in risk stratification for OSCC. Among these factors, tobacco use stands out as a major risk factor, with patients who use tobacco having worse survival. History of tobacco use is used as a factor to escalate adjuvant treatments in some head and neck cancer clinical trials^2^. Whole exome sequencing data has shown increased mutational burden in head and neck SCC (HNSCC) patients who use tobacco^3^; however, very little is known about the epigenomic or gene expression landscape of tobacco use in OSCC. Epigenetic changes play an important role in early OSCC, with hyper- or hypo-methylation of critical genes causing an alteration of gene expression, contributing to carcinogenesis and genomic instability^4^. Epigenetic dysregulation is one of the most frequent events occurring early in oral carcinogenesis^4^. While epigenetic and gene expression studies in OSCC patients^4–14^, including our own studies^5,6^, have highlighted specific genes, none of these studies have focused on tobacco-specific epigenetic or gene expression changes.

In this study we hypothesized that tobacco use imparts epigenome or gene expression-specific changes that, when combined with salient histologic, clinical and demographic data, could be used as a biomarker to predict disease outcome. To test our hypothesis and develop our composite molecular and non-molecular biomarker risk score, we took a multi-omic approach to analyze methylation array and RNASeq data from OSCC patients in The Cancer Genome Atlas (TCGA). We identified the significant genome-wide epigenetic and gene expression changes in this publicly available cohort, and validated our findings in an internal cohort of OSCC patients prospectively enrolled at our institution. We performed functional network analyses to determine the biologic, cellular and molecular processes that were impacted by the dysregulated genes in smokers. We then used deep learning models to derive histologic markers that were predictive of tobacco use. Lastly, we determined its predictive performance of the multi-omic epigenetic, expression, and histologic biomarker. We showed that while tobacco use and clinicopathologic factors alone were inaccurate in predicting mortality, the multi-omic biomarker, when combined with clinicopathologic factors, was highly predictive of mortality.

## Results

### Patient cohort characteristics

The TCGA cohort (*n* = 257) with complete clinicopathologic data and tobacco pack-year use included patients with OSCC at all stages (I-IV). Table 1 details their demographic and clinicopathologic characteristics. Median and mean age were both 61 years. 64.2% were male; 87.55% were white, 5.45% were Black, 3.5% were Asian, and 4.67% identified as Hispanic ethnicity. The racial and ethnic distributions are similar to previous oral cavity cancer cohorts^15^. Clinical stage was as follows: 3.5% stage I, 24.51% stage II, 22.96% stage III, and 45.91% stage IV. 62.65% of the cohort were never smokers and 37.35% of the cohort had a ≥ 10 pack year history. Fourteen oral SCC patients with a < 10 pack year history were excluded from the analysis to create two divergent cohorts of life time non-smokers and heavy smokers for epigenetic analysis. There were 88 patients (34.24%) who died by 5 years after diagnosis; 57 of these patients had a ≥ 10 pack year history, with smokers significantly more likely to die than nonsmokers **X**^2^ (1, *n* = 257) = 4.3106, *p* = 0.038.

**Table 1:** TCGA patient demographics and clinicopathologic characteristics

	TCGA (*n* = 257)	Internal cohort (*n* = 40)
Tumor location		
Tongue	114 (44.36%)	14 (35%)
Floor of mouth	48 (18.68%)	3 (7.5%)
Alveolar ridge (maxilla/mandible)	17 (6.61%)	21 (52.5%)
Buccal mucosa	16 (6.23%)	1 (2.5%)
Hard palate	4 (1.56%)	0
Lip	3 (1.17%)	1 (2.5%)
Oral cavity, NOS	55 (22.4%)	0
Sex		
Female	92 (35.80%)	23 (57.5%)
Male	165 (64.20%)	17 (42.5%)
Age		
Median; mean	61; 61.48	65; 67.03
Race		
White	225 (87.55%)	33 (82.5%)
Black	14 (5.45%)	2 (5%)
Asian	9 (3.50%)	5 (12.5%)
Other	9 (3.50%)	0
Ethnicity		
Hispanic	12 (4.67%)	4 (10%)
Non-Hispanic	245 (95.33%)	36 90%)
Tobacco use		
Never smoker	96 (37.35%)	15 (37.5%)
Current/previous smoker; [≥10 pack years]	161 [161] (62.65%)	25 (62.5%)
Alcohol use		
No	92 (35.80%)	4 (22.22%)
Yes	165 (64.20%)	14 (77.78%
Survival at 5 years		
Alive	202 (78.60%)	36 (90%)
Dead	88 (34.24%)	4 (10%)
Tumor grade		
G1	38 (14.79%)	22 (55%)
G2	161 (62.64%)	15 (37.5%)
G3	55 (21.40%)	3 (7.5%)
Margin status		
Negative	180 (70.04%)	37 (92.5%)
Close (<5 mm)/positive	65 (25.29%)	3 (7.5%)
Perineural invasion		
No	95 (36.96%)	11 (64.71%)
Yes	110 (42.80%)	6 (35.29%)
AJCC clinical stage		
Stage I	9 (3.50%)	13 (32.5%)
Stage II	63 (24.51%)	4 (10%)
Stage III	59 (22.96%)	9 (22.5%)
Stage IV	118 (45.91%)	14 (35%)

The table details the characteristics of the cohort.

*AJCC* American Joint Committee on Cancer, *NOS* not otherwise specified, *TCGA* The Cancer Genome Atlas

The internal cohort contained 40 patients. Demographics are also listed in Table 1. Mean age of the cohort was 67.03. 42.5% were male and 82.5% were white. Breakdown of smokers to never smokers were similar to TCGA, with 62.5% of the internal cohort being current or previous tobacco users.

### Methylation array analysis reveals differentially methylated genes with heavy tobacco use that is independent of cancer stage

**Fig. 1:**
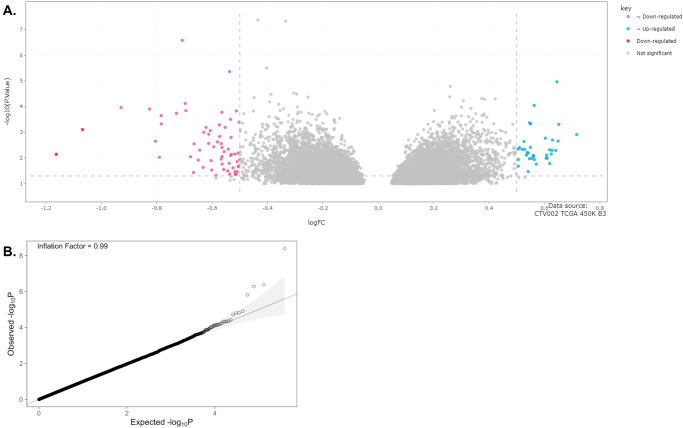
TCGA methylation analysis results. **A** Volcano plot of batched corrected data. Only differentially methylated sites with unadjusted *p* < 0.1 are included. Unadjusted *p* < 0.05 and log fold change > +/−0.5 are considered significant. **B** QQ plot of the batch corrected data, which compares the expected to observed -log_10_P, and demonstrates an inflation factor = 0.99.

We compared methylation between never smokers and ≥10 pack years, controlling for age, sex, and cancer stage (I-IV) as covariates. Figure 1A illustrates a volcano plot of the differentially methylated sites with an unadjusted *p* < 0.1 (points in grey). Sites with an unadjusted *p* < 0.05 and log fold change >±0.5 are further highlighted in color. Data integrity is observed with a QQ plot of the batch corrected data, which compares the expected to observed -log_10_P, and demonstrates an inflation factor = 0.99 (Fig. 1B), signifying integrity of the epigenome wide association study (EWAS) data even before batch correction. Differentially methylated genes were calculated between never smokers and heavy smokers (≥10 pack year history). Table 2 details the epigenome wide significant genes between never smokers and heavy smokers. Table 3 similarly lists the differentially methylated genes between never smokers and heavy smokers, after controlling for the following covariates: age, sex, and clinical stage. Interestingly, the top three genes, *GNG12, GPR15*, and *GDNF*, which meet the cut off of adjusted *p* < 0.05, are the same in both Tables 2 and 3, indicating that the significant methylation differences in these three genes are driven by tobacco use alone. *GNG12* and *GPR15* have not previously been associated with head and neck cancer, and there are no published preclinical or clinical studies linking these genes to head and neck carcinogenesis and smoking. *GDNF* altered expression, but not epigenetic changes, has been implicated in head and neck cancer perineural invasion^16^, and high *GDNF* expression has been linked to poor survival in one cohort, but the results were not replicated in a larger TCGA cohort^17^.

**Table 2:** Differentially methylated sites between never smokers and heavy smokers

Name	Chr	Position	Gene	logFC	Average methylation	*p* value	Adjusted *p* value	*t*	*B*
cg25189904	chr1	68299493	GNG12	−0.342664459	−1.636517277	4.09405E−09	0.000790225	−6.172311411	6.212338121
cg19859270	chr3	98251294	GPR15	−0.385081137	2.231523764	4.24774E−07	0.034611858	−5.243185017	3.324913923
cg18121355	chr5	37840438	GDNF	−0.655269498	−2.421679015	5.37958E−07	0.034611858	−5.193024096	3.177378538
cg09147586	chr22	50612372	PANX2	0.352194966	2.438202924	1.54218E−06	0.074417064	4.965187366	2.519181147
cg04685302	chr8	142229056	SLC45A4	0.252638359	1.957717487	1.26816E−05	0.429068862	4.485590508	1.201443989
cg14410227	chr14	101488345	MIR411	−0.477543929	2.332442178	1.53914E−05	0.429068862	−4.439666261	1.080356798
cg26986447	chr17	27406701	MYO18A	0.25669182	2.662190389	1.55606E−05	0.429068862	4.437062444	1.07351905
cg25758242	chr6	29427011	OR2H1	−0.54772037	1.806826854	1.92321E−05	0.464017196	−4.386405697	0.941086683
cg20242392	chr7	137439258	DGKI	−0.345902646	−2.171089527	3.7872E−05	0.701571332	−4.221400213	0.517669045
cg03043296	chr15	83349420	AP3B2	−0.330431301	−0.878405168	4.46971E−05	0.701571332	−4.180328278	0.414199625

Gene position, name, methylation fold-change, and *p* values are shown. The differentially methylated genes are calculated based on smoking status alone. The top three genes, GPR15, GNG12, and GNDF, meet the adjusted *p* value cutoff of 0.05.

**Table 3:** Differentially methylated sites between never smokers and heavy smokers (covariates included)

Name	Chr	Position	Gene	logFC	Average methylation	*p* value	Adjusted *p* value	*t*	*B*
cg19859270	chr3	98251294	GPR15	−0.434606206	2.231523764	4.10742E−08	0.004476246	−5.727064309	4.451074695
cg25189904	chr1	68299493	GNG12	−0.334421204	−1.636517277	4.63816E−08	0.004476246	−5.702718377	4.378415176
cg18121355	chr5	37840438	GDNF	−0.707730828	−2.421679015	2.60553E−07	0.016763793	−5.349255756	3.344502853
cg20242392	chr7	137439258	DGKI	−0.403259325	−2.171089527	3.10395E−06	0.149779651	−4.812203262	1.855264302
cg14410227	chr14	101488345	MIR411	−0.536855975	2.332442178	4.32294E−06	0.166880893	−4.737236349	1.65588177
cg00709541	chr19	58511273	ZNF606	0.645214821	2.013788554	1.07577E−05	0.346071331	4.526401662	1.107045099
cg04685302	chr8	142229056	SLC45A4	0.259981024	1.957717487	1.62658E−05	0.448513958	4.428433799	0.858165319
cg08879684	chr13	46962963	C13orf18	−0.305172175	0.491846344	2.95132E−05	0.627261888	−4.284427999	0.499640616
cg25702001	chr8	94770263	TMEM67	−0.293899259	1.558728718	3.25564E−05	0.627261888	−4.26036739	0.440602093
cg19203203	chr7	106508855	PIK3CG	−0.387340658	2.43232467	3.31429E−05	0.627261888	−4.255979495	0.429862317

Gene position, name, methylation fold-change, and *p* values are shown. The differentially methylated genes are calculated based on smoking status, while taking into account the following covariates: age, sex, and clinical stage. The top three genes match those of Table 2: *GPR15, GNG12*, and *GNDF*. These similar findings indicate that the differential methylation changes are driven by smoking status alone.

Validation of the three differentially methylated genes was performed using the internal cohort, after batch correction and controlling for age, sex, and clinical stage. The three genes showed methylation change at the indicated methylation site, with the logFC (log fold change) and *p*-value as follows: *GNG12* (cg25189904, logFC = −0.617, *p* = 0.05), *GPR15* (cg08375941, logFC = −0.741, *p* = 0.07, and *GDNF* (cg05330056, logFC = −0.868, *p* = 0.01). The direction of the logFC values (negative value) matched those of the TCGA cohort (Tables 2 and 3). While there were only 3 significant genes that met or approached the stringent adjusted *p*-value cutoff of 0.05, larger cohorts (of over 21,000 samples) examining epigenetic dysregulation in smoking and carcinogenesis have produced an equally limited number of differentially methylated genes^18^.

### RNASeq analysis adds to the multi-omic biomarker panel of tobacco use in oral SCC

**Fig. 2:**
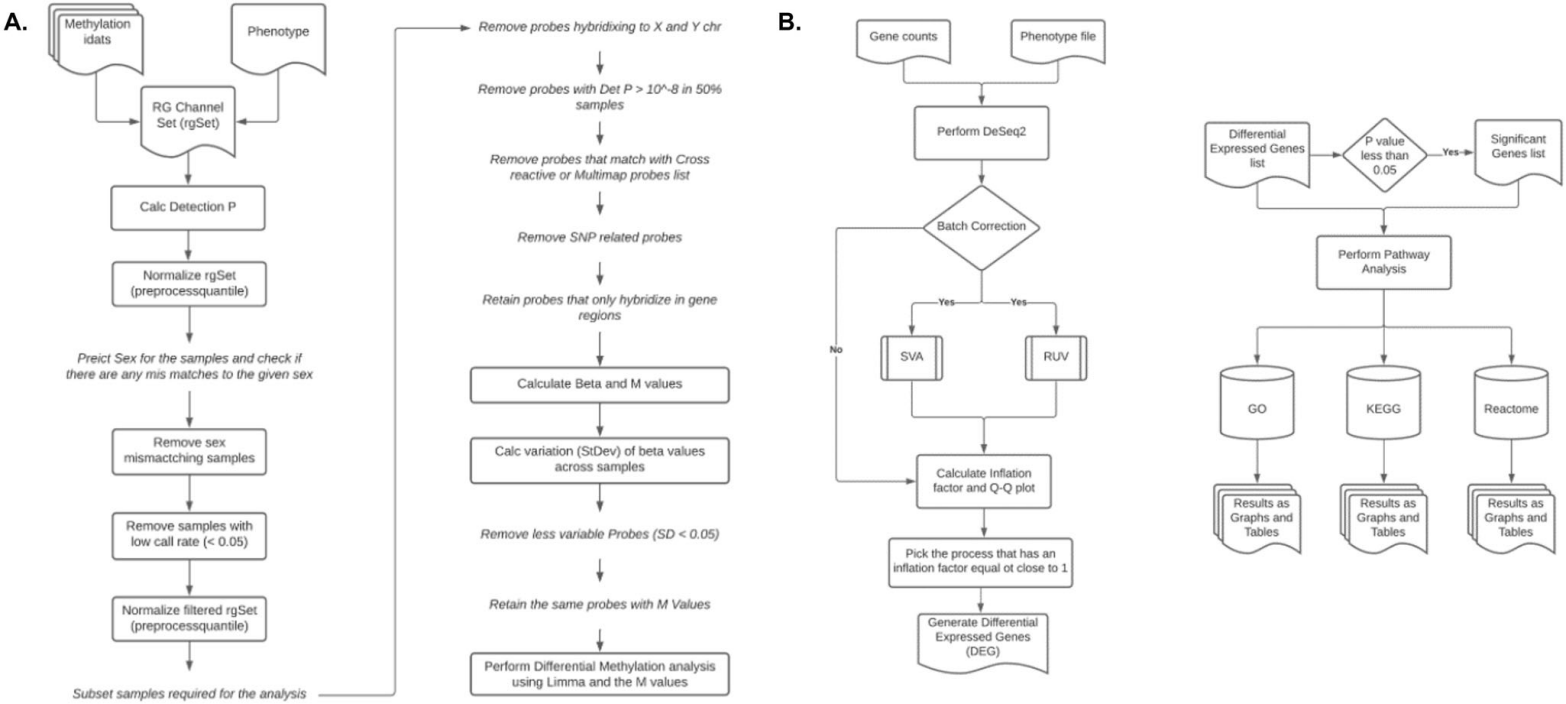
Methylation and RNA Seq array work flow. The analysis steps for the array data from the TCGA cohort are shown, with (**A**) representing the methylation array workflow and (**B**) representing the RNA Seq workflow.

In a parallel analysis (Fig. 2) we focused on differential gene expression using available RNASeq results. The cohorts were divided similar to the epigenetic analysis in that never smokers were compared against heavy smokers (≥10 pack year use). The goal of the analysis was not to match the significant epigenetic marks to their complementary RNASeq marks, as our previous multi-omic biomarker studies in OSCC have demonstrated that DNA hypo- and hypermethylation can be linked to gene under- or over-expression, depending on the gene^15^. Rather, the purpose of the additional RNASeq analysis was to produce additional expression biomarkers that are specific to tobacco use. This list of differential expressed genes added to the 3 differentially methylated genes from the EWAS. Table 4 details the 22 differentially expressed genes between never smokers and heavy smokers. These genes met the adjusted *p* value = 0.05 cut off. When covariates (age, sex, and clinical stage) were taken into account, there were 13 differentially expressed genes (Table 5). Twelve of the 13 differentially expressed genes matched the significant genes in Table 4 (i.e., with the exception of *FMO3*, the remaining 12 genes were differentially expressed based on smoking status alone, regardless of covariates). Six of the 13 genes reaching statistical significance in our cohort have been evaluated in head and neck cancer. *SCG5* expression has been used as part of a nine-gene panel to predict OSCC prognosis^19^. Our group has shown that *NTRK1* is critical to OSCC perineural invasion and metastasis^20^. *CD96* is an immune regulatory checkpoint molecule that is significantly increased in OSCC tissue^21^. *BMP6* over-expression is associated with OSCC bone invasion^22^. *TFPI2* hypermethylation is associated with worse overall survival in OSCC patients from the TCGA database in a study focused on epigenetic dysregulation of tumor suppressor genes^23^. *RYR3* RNA levels is shown to be correlated to survival of head and neck SCC^24^. The remaining 7 genes have not been meaningfully evaluated in head and neck SCC studies.

**Table 4:** Differentially expressed sites between never smokers and heavy smokers

ENTREZID	Symbol	log2 fold change	*p* value	Adjusted *p* value	*q* value	Base mean	Gene name
3494	IGHA2	1.344049508	6.41808E−10	9.16694E−06	7.83975E−06	9603.66904	Immunoglobulin heavy constant alpha 2 (A2m marker)
107075270	MTCO3P12	−1.516510015	4.22836E−07	0.003019683	0.002582492	423.235845	MT-CO3 pseudogene 12
10225	CD96	0.392068376	1.01801E−06	0.004846728	0.004145018	330.2502028	CD96 molecule
654	BMP6	−0.680414865	2.26935E−06	0.008103268	0.006930074	150.7830131	Bone morphogenetic protein 6
2039	DMTN	0.572253662	2.95121E−06	0.008430439	0.007209878	914.0441746	Dematin actin binding protein
4914	NTRK1	−0.742693693	5.14034E−06	0.011476223	0.009814692	72.86184756	Neurotrophic receptor tyrosine kinase 1
203260	CCDC107	−0.515296032	5.62442E−06	0.011476223	0.009814692	246.4800828	Coiled-coil domain containing 107
7980	TFPI2	1.214487263	6.44709E−06	0.011510474	0.009843985	655.6201679	Tissue factor pathway inhibitor 2
8685	MARCO	−0.785274416	1.21043E−05	0.019209524	0.016428364	424.1220125	Macrophage receptor with collagenous structure
6263	RYR3	−0.664688897	1.84164E−05	0.025278552	0.021618716	240.5153842	Ryanodine receptor 3
157869	SBSPON	−0.763574801	1.99155E−05	0.025278552	0.021618716	151.6477397	Somatomedin B and thrombospondin type 1 domain containing
6123	RPL3L	−0.687934931	2.1238E−05	0.025278552	0.021618716	297.0342124	Ribosomal protein L3 like
2820	GPD2	0.25609952	3.57242E−05	0.038546983	0.03296614	3520.468008	Glycerol-3-phosphate dehydrogenase 2
29952	DPP7	0.280376763	4.07581E−05	0.038546983	0.03296614	3881.966618	Dipeptidyl peptidase 7
79870	BAALC	−0.711363092	4.22849E−05	0.038546983	0.03296614	276.1541724	BAALC binder of MAP3K1 and KLF4
27132	CPNE7	0.648937938	4.40293E−05	0.038546983	0.03296614	195.2831619	Copine 7
30008	EFEMP2	0.285241986	4.58796E−05	0.038546983	0.03296614	1690.66168	EGF containing fibulin extracellular matrix protein 2
6447	SCG5	0.485609154	5.16072E−05	0.040950336	0.035021534	235.5603169	Secretogranin V
338773	TMEM119	0.398588004	5.7323E−05	0.043091815	0.03685297	761.9948457	Transmembrane protein 119
7016	TESK1	−0.475268342	6.8049E−05	0.04859722	0.0415613	2098.770581	Testis associated actin remodelling kinase 1
3512	JCHAIN	0.547023725	7.56311E−05	0.051439972	0.043992478	4059.443223	Joining chain of multimeric IgA and IgM
283349	RASSF3	−0.262656085	8.71736E−05	0.056595482	0.048401572	1321.040087	Ras association domain family member 3

Twenty-two genes meet the adjusted *p* value <0.05 cutoff.

**Table 5:** Differentially expressed sites between never smokers and heavy smokers (covariates included)

ENTREZID	Symbol	log2 fold change	*p* value	Adjusted *p* value	*q* value	Base mean	Gene name
3494	IGHA2	1.582569279	5.16707E−12	7.38012E−08	6.63956E−08	9603.66904	Immunoglobulin heavy constant alpha 2 (A2m marker)
6447	SCG5	0.61399688	8.78415E−07	0.006273199	0.005643711	235.5603169	Secretogranin V
6123	RPL3L	−0.822211866	1.63881E−06	0.007802393	0.007019458	297.0342124	Ribosomal protein L3 like
4914	NTRK1	−0.795379452	3.53092E−06	0.012608024	0.011342865	72.86184756	Neurotrophic receptor tyrosine kinase 1
10225	CD96	0.386615523	5.89036E−06	0.016250134	0.014619505	330.2502028	CD96 molecule
654	BMP6	−0.686799891	6.82635E−06	0.016250134	0.014619505	150.7830131	Bone morphogenetic protein 6
7980	TFPI2	1.208548544	1.98117E−05	0.040084146	0.03606188	655.6201679	Tissue factor pathway inhibitor 2
2328	FMO3	−0.857213206	2.35551E−05	0.040084146	0.03606188	176.1881303	Flavin containing dimethylaniline monoxygenase 3
30008	EFEMP2	0.307877139	2.52578E−05	0.040084146	0.03606188	1690.66168	EGF containing fibulin extracellular matrix protein 2
6263	RYR3	−0.680812338	3.35145E−05	0.046878613	0.042174552	240.5153842	Ryanodine receptor 3
2039	DMTN	0.534149743	3.79448E−05	0.046878613	0.042174552	914.0441746	Dematin actin binding protein
2820	GPD2	0.272595321	3.93855E−05	0.046878613	0.042174552	3520.468008	Glycerol-3-phosphate dehydrogenase 2
79870	BAALC	−0.761868857	4.4826E−05	0.049249932	0.04430792	276.1541724	BAALC binder of MAP3K1 and KLF4

Thirteen genes meet the adjusted *p* value <0.05 cutoff. Of these, 12 are replicated in Table 4, where covariates are not considered. These 12 genes are used in our biomarker calculation.

Validation with the internal cohort was challenging as our gene expression data was derived from the HT-12 Gene Expression Array representing a different platform from the RNASeq platform. However, we demonstrated that 9 of the top 13 differentially expressed genes also showed differential expression in the internal cohort. These 9 genes were: *SCG5, RPL3L, CD96, BMP6, FMO3, EFEMP2, RYR3, GPD2*, and *BAALC*.

### Functional pathway analysis of the gene markers in heavy smokers

**Fig. 3:**
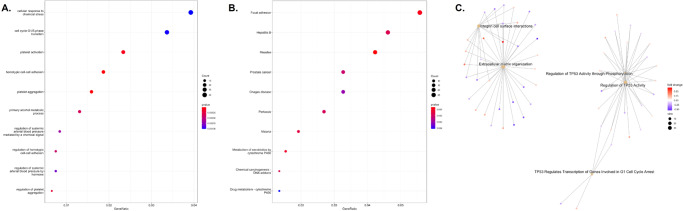
Functional pathway analysis of RNASeq biomarkers. **A** GO BP top 10 dysregulated pathways. **B** Top 10 dysregulated KEGG pathways. **C** Top 5 Reactome gene sets.

We performed functional pathway analysis of the differentially expressed genes using: 1) GO pathways that were linked to the candidate genes aggregated by gene ontology category (i.e., biological process, cellular compartment, molecular function), 2) KEGG pathways, and 3) Reactome pathways. Figure 3A shows the top 10 perturbed GO biological process pathways, with many of the pathways related to platelet activation, platelet aggregation, cell-cell adhesion, and blood pressure control. KEGG pathway analysis similarly demonstrates that the focal adhesion pathway is the top dysregulated pathway (Fig. 3B). Lastly, Reactome analysis shows that integrin cell surface interactions, extracellular matrix organization, TP53 regulation are the top dysregulated pathways (Fig. 3C).

**Fig. 4:**
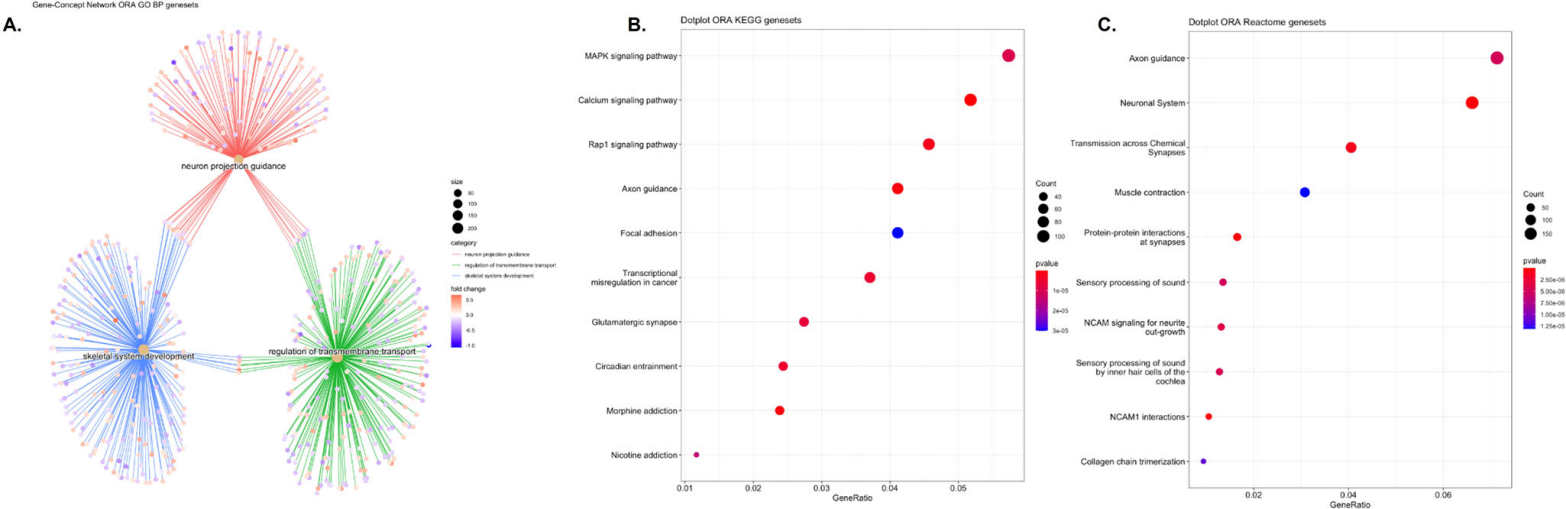
Functional pathway analysis of methylation biomarkers. **A** GO BP gene concept network. **B** Top 10 dysregulated KEGG pathways. **C** Dot plot of ORA Reactome gene sets.

Functional pathway analysis was similarly performed for the differentially methylated genes to determine significant gene networks among heavy smokers using 1) GO biologic process pathways, 2) KEGG pathways, and 3) Reactome pathways. Figure 4 details the top perturbed pathways. Overall, neuron projection guidance, axon guidance, axonogenesis, synaptic signaling and neuronal system gene sets, all which relate to perineural invasion and neuron cross talk, were consistently dysregulated across all three functional network analysis platforms. Similar to the functional network results of the gene expression data, focal adhesion was one of the top dysregulated pathways in the KEGG analysis, demonstrating that the methylation and expression biomarkers were responsible for similar gene networks. Within our KEGG analysis of the methylation data, morphine addiction was one of the dysregulated pathways. We have recently shown in a biomarker study that the morphine addiction pathway is a top pathway controlled by methylation, and portends a poor prognosis even in early stage OSCC patients in the TCGA cohort^15^.

### Histologic modeling

**Fig. 5:**
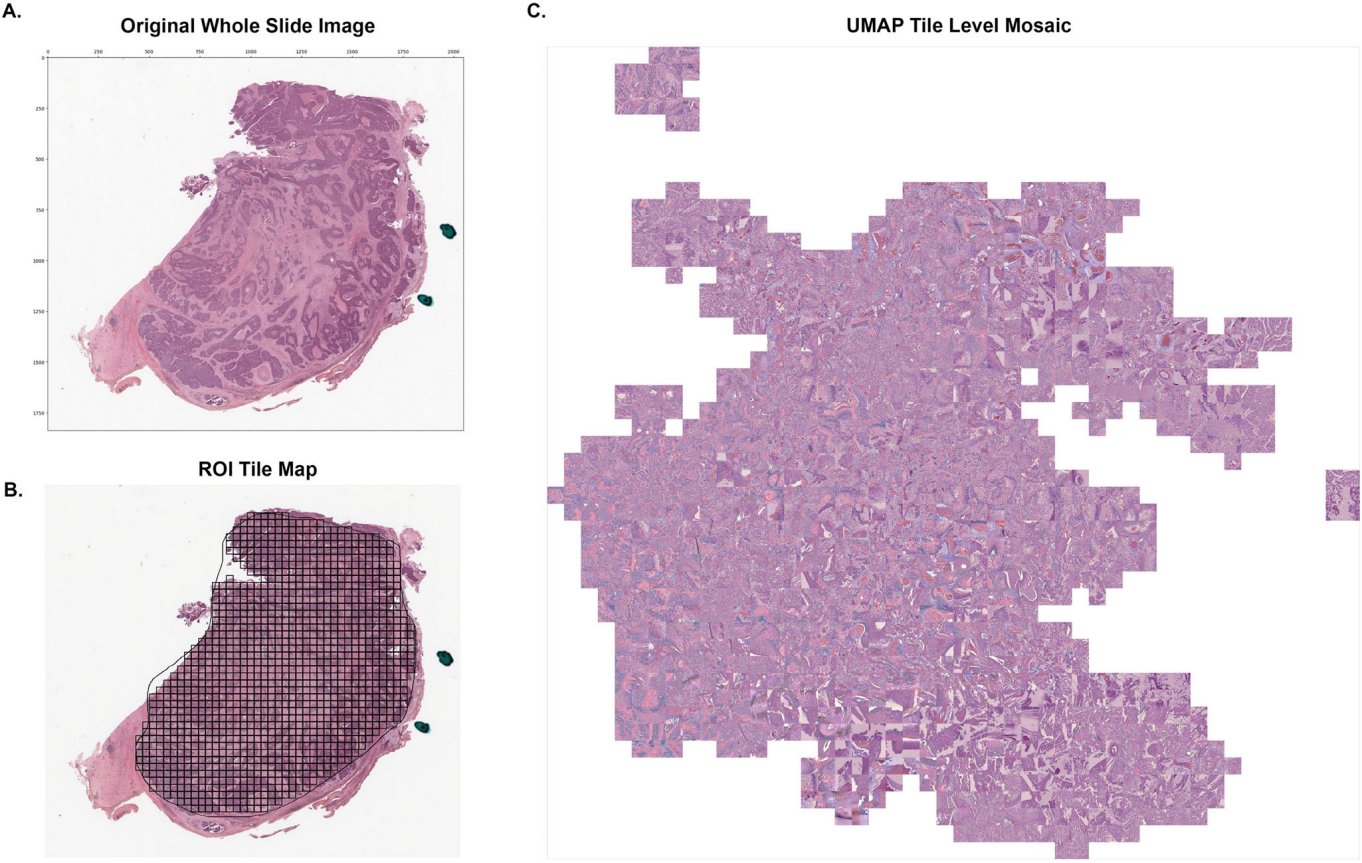
Digital histopathology analysis with a deep learning model designed to predict patient smoking status. **A** Whole Slide Images from 203 TCGA hematoxylin and eosin stained histopathology slides served as training data for a deep learning model constructed with the Slideflow pipeline. **B** Expert pathologists annotated regions of interest (ROI) on each WSI. Within each ROI, the WSIs are divided into tiles of size 299 pixels × 299 pixels. Tiles underwent stain normalization and augmentation prior to model training. **C** UMAP of the post-convolution layer activations from all images in the validation set. Plotted tiles are a subset of all image tiles within the validation set.

**Fig. 6:**
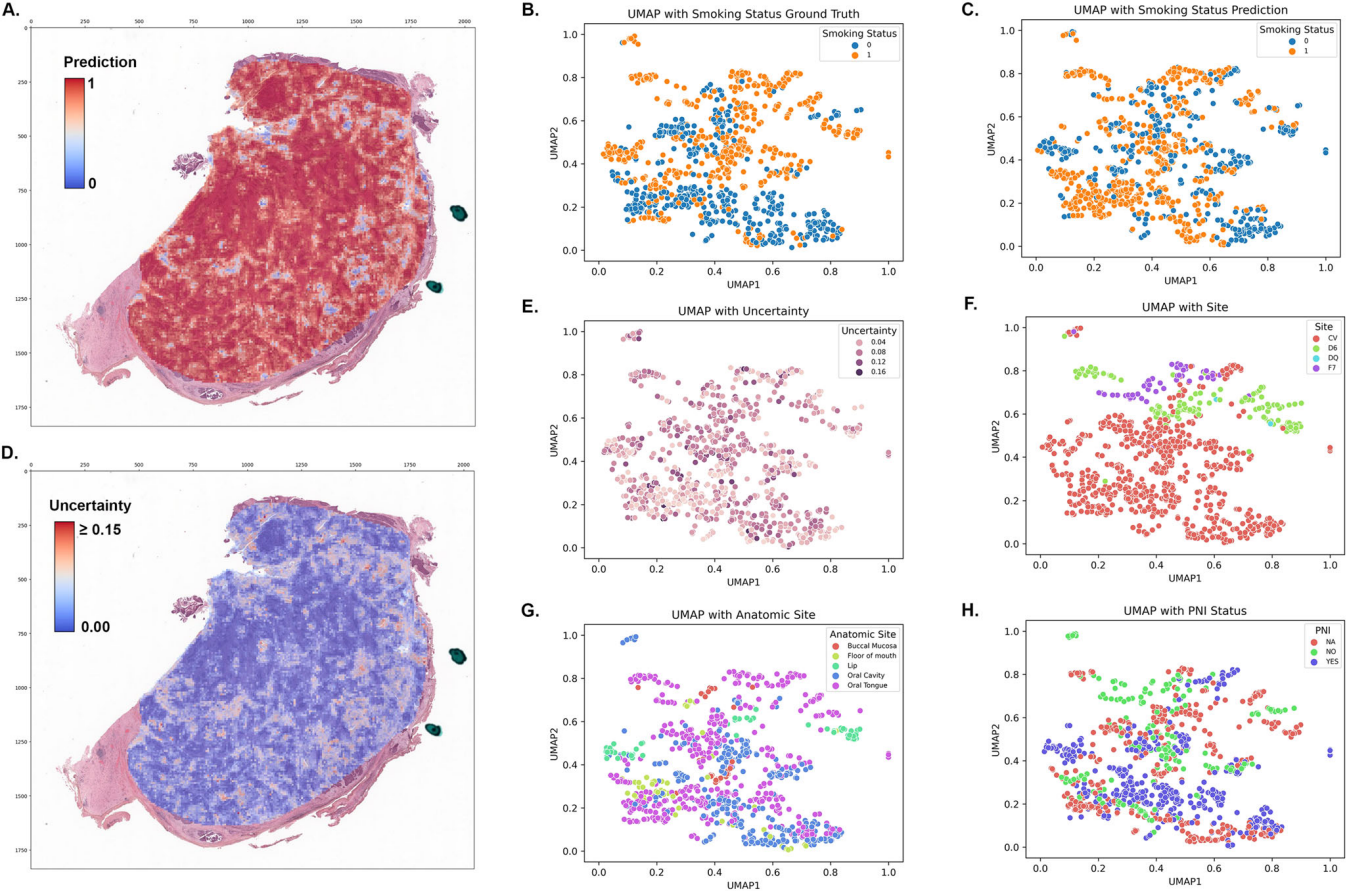
Deep learning model explainability analysis. **A** Heat map of the model’s logit score assigned to a given location within the image’s ROI. **B** UMAP of the post-convolutional layer activations from all images in the model’s validation set with a label of the model’s smoking status prediction (1- heavy smoker, 0- non-smoker). **C** UMAP in **B** labeled with the ground truth smoking status prediction. **D** Heat map of the model’s uncertainty quantification of the outcome prediction assigned to a given location within the image’s ROI. **E** UMAP in **B** labeled with the uncertainty quantification. **F** UMAP in **B** labeled with the TCGA donating site. **G** UMAP in **B** labeled with anatomic site. **H** UMAP in **B** labeled with perineural invasion status (PNI).

We generated patient level histology scores as described. The model predicted the likelihood that a patient with the specific histologic features was a smoker, and the likelihood of mortality in five years after diagnosis. A deep learning model was trained on 215 pathologist-annotated WSIs from TCGA (Figs. 5 and 6) to predict smoking status and vital status. A positive smoking status included patients with a ≥ 10 pack-year smoking history and a negative smoking status included patients with no history of smoking or tobacco use. After training in a 3-fold cross validation with site-preservation, we extracted pre-logit scores from the final activation layer in the validation set of each k-fold. The pre-logit scores then constituted a prediction score for the outcome of interest based on the deep learning model’s ability to characterize each outcome and could be integrated into additional multivariate analysis. The models predicting smoking status achieved patient-level AUROCs of 0.62, 0.49, 0.52 and PPV of 0.69, 0.65, and 0.66 for k-fold1, k-fold2, and k-fold3, respectively (Supplementary Table 3). Models predicting vital status at five years had lower performance with patient-level AUROCs of 0.48, 0.54, 0.53 and PPV of 0.66, 0.61, and 0.71 in k-fold1, k-fold2, and k-fold3, respectively (Supplementary Fig. 1). All model statistics including AUROC, AUPRC, PPV, NPV, sensitivity, and specificity are shown in Supplementary Table 3.

To better understand the histologic features detected by the model and their relationship to smoking status, we trained a model with 183 images in the training set without cross-validation and generated a UMAP plot from post-convolutional layer activations for each tile across all slides. Each dot on the UMAP plot represented the tile nearest to the centroid from each slide. Points were then labeled in each plot by outcome, uncertainty, or logit-score. Without cross validation or additional measures to address site specific biases, the model achieved an AUROC of 0.67.

### Generation of mortality risk score based on multi-omic biomarker

We generated a mortality risk score based on 9 non-molecular clinicopathologic factors (age, sex, race, ethnicity, alcohol use, clinical stage, histologic grade, presence of perineural invasion, presence of lymphovascular invasion, and margin status), 3 methylation biomarkers, 13 expression biomarkers, and the logit score from histologic deep learning models. Risk score generation was performed after validation of the multi-omic biomarkers in the two cohorts. These biomarkers had been developed by dichotomizing patients as never smokers vs. ≥10 pack year history. In contrast, the risk score dichotomized patients based on survival status. For the 3 methylation biomarkers, methylation percentage was classified as tertiles (cutoff values at 0.33 and 0.75) as we have previously done^15^, with a stringent requirement for the methylation index to change from a lower to higher tertile to be considered hypermethylated, and vice versa to be considered hypomethylated. Inverse weight of the tertiles was used since the associations decreased risk with hypermethylation in our models. Since some of the differentially expressed genes were positively correlated with smoking and some negatively correlated with smoking, these genes were divided into two sets and genes with negative correlation were inverse coded, for interpretability. A gene expression fold change of 1.5 was considered significant. The ability of smoking status alone to predict five-year mortality was low. When pack years were considered, a pack year status of >10 pack years was only accurate in predicting 5-year mortality with a c-statistic = 0.5378. Smoking status of current vs. former or never smokers was accurate in predicting 5-year mortality with a c-statistic = 0.5014. The combination of the clinical factors (age, sex, smoking pack years), histological modeling and the 16 gene targets was able to predict 5-year mortality with a c-statistic = 0.9409.

## Discussion

In this study we interpreted epigenomic and gene expression data from a publicly available OSCC cohort (TCGA) and validated our findings in an internal OSCC cohort to arrive at 3 epigenomic and 13 expression biomarkers of tobacco use that have prognostic ability in determining OSCC outcome. We combined our gene features with a histologic analysis to produce a multi-omic biomarker that has not previously been done in OSCC. The study identifies gene features and histologic characteristics altered by tobacco use that are independent of other clinical covariates. To our knowledge, our biomarker panel using these 16 gene features, histologic characteristics, and clinical covariates is of the highest accuracy in predicting 5-year mortality of biomarker studies to date^15,25,26^.

Completion of the human genome project at the turn of the century coupled with rapidly advancing gene sequencing and array technology facilitated a surge in biomarker studies in cancer patients. In some cancers, the results have translated into clinically robust biomarker panels and discovery of precise anti-cancer drugs^27,28^. However, oral cancer treatment and prognosis have remained stagnant. In fact, worldwide OSCC incidence is increasing^1^. Tobacco use remains a significant risk factor for OSCC development. However, no multi-omic studies have been performed to identify the tobacco-specific perturbations in OSCC patients that might have prognostic significance, and no studies have combined genomic and histologic signatures to predict mortality risk in OSCC.

A number of genes belonged to differentially perturbed KEGG pathways that were associated with heavy smoking. The gestalt of pathway analyses, based on the top ten most differentially perturbed pathways that harbored genes with smoking-associated CpG, were involved in cancer and immune function, including pathogen response. It warrants mention that KEGG annotations were curated from literature that largely predates more recent attempts at a function-based, rather than disease-centric nomenclature, the latter which can result in challenges to interpretation of the actual mechanistic functions of the genes. For example, in the case of the top three pathways identified in through the KEGG database analysis, there were 24 genes that were differentially expressed in the focal adhesion pathway; 5 of these 24 genes also contributed to the hepatitis B pathway and 6 of the 24 genes contributed to the measles pathway, with 4 genes being shared among all three pathways (i.e., *BAD*, *PIK3R3*, *JUN*, *MAPK8*). The “hepatitis B” and “measles” pathways are disease-centric gene pathways; mechanistically, the genes function across a wide range of cellular processes, including focal adhesion, and are well-recognized to be perturbed in cancer. We speculate that the pathway names may be misnomers in the context of the current study.

Previous epigenomic biomarker studies have instead focused on tobacco users without a history of cancer. One study conducted an EWAS using the Illumina 450 K array on current, former and never smokers in a German cohort totaling 1793 participants. DNA methylation levels in former smokers were found to be similar to never smokers with more time elapsed after tobacco cessation. Methylation specific protein binding patterns were observed for cg055759 in *AHRR* in current smokers. *AHRR* is a known tumor suppressor gene. The study also identified *GNG12* as a hypermethylated gene in current smokers, which matches our findings in OSCC patients who were heavy smokers. While the study identified a total of 187 smoking-specific CpG sites that had significant changes in two separate cohorts, the biologic samples used were blood samples and not tissue samples, and coupled with the fact that participants were smokers without a cancer history, no additional conclusions could be drawn on the correlation between these methylation changes and tissue-specific carcinogenic changes^29^.

Epigenomic analysis of the TCGA cohort discovered 3 gene markers that were validated in our internal cohort: *GNG12, GPR15*, and *GDNF*. Guanine nucleotide‐binding protein subunit gamma‐12 (GNG12) acts as a modulator of a number of transmembrane signal pathways, several of which have been demonstrated to play a role in cancer^30^. Both increased^31,32^ and decreased^33^ GNG12 expression have been reported in different cancers, while the *GNG12* gene has not been studied for its role in oral cancer. We found decreased methylation of cg25189904 in TCGA samples of patients who were heavy smokers compared to those collected form never smokers, an association also observed previously^34,35^. Decreased methylation of CpG site cg25189904 of the *GNG12* gene, which is located in the promoter region transcription start site (proximal 1500 base pairs of the *GNG12* promoter), is speculated to result in the increased expression of *GNG12* and increased protein levels of GNG12. *GPR15* is a G-protein coupled receptor that acts a chemokine receptor; it is suggested to play a role of immunomodulatory perturbation in colorectal^36^ and gastric^37^ cancer, and is also found to harbor differentially methylated CpG sites influenced by smoking^38,39^. We found decreased methylation of cg19859270 in TCGA samples of patients who were heavy smokers compared to those collected form never smokers. Decreased methylation of CpG site cg19859270, which is located in the first exon of the gene^40^ and is considered to be part of the promoter region, is correlated with increased expression of *GPR15* and protein levels of GPR15^41^. *GDNF* is a glial cell derived neural growth factor. Increased GNDF has been reported to play a role in colon cancer metastasis and colon cancer cell migration; it also plays a role in other cancers^42^ including head and neck cancer^16^, and is influenced by smoking behavior^43,44^. We found decreased methylation of cg18121355 in TCGA samples of heavy smokers compared to those collected form never smokers. Methylation of cg18121355, which is located in the promoter region transcription start site (proximal 1500 base pairs of the *GDNF* promoter), is speculated to increase expression of *GNDF*.

A review of the epigenetic studies in OSCC identifies tobacco consumption and the resultant formation of covalent bonds between the carcinogens in tobacco with DNA, leading to DNA damage, as a mechanism for global DNA hypomethylation^45^. Gene specific hypomethylation in response to tobacco is seen as a method of activating oncogenes in the process of genomic integrity loss during oral carcinogenesis^46^. At the same time, several tumor suppressor genes are hypermethylated in response to tobacco use. *CDKN2A (p16), CDH1*, and *P15* have been identified in multiple studies using OSCC samples, including our own, as being hypermethylated in early oral carcinogenesis. In terms of concurrent tobacco and alcohol use, clinical studies have had difficulty isolating the effects of tobacco and alcohol use alone, as patients tend to use both tobacco and alcohol together, with both being confounders for each other. We have previously defined a methylation biomarker of five genes, *APC, CDH1, MGMT, p15* and *p16*, in which all five genes were hypermethylated in the saliva of OSCC patients^5^. This gene panel was subsequently adapted in follow up studies by other groups, including those examining the epigenetic effects of tobacco and alcohol. In a separate publication, *p16, CDH1, MGMT, APC*, and *DAPK* were shown to be hypermethylated in OSCC patients with tobacco and alcohol use habits^47^. However, these gene targets were shown across multiple studies to be hypermethylated in early oral carcinogenesis regardless of smoking status.

While methylation array analysis of the TCGA cohort only produced 3 gene candidates that were validated in our internal cohort, other similar studies have produced an equally small number of biomarkers even with large cohorts. For example, an array study with 21,000 blood samples and 7700 tissue samples from TCGA explored a subset of 495 patients with head and neck SCC and found 4 significant expression markers that were linked to tobacco mutational signatures: *NFE2l2, RMND5A, SLC44A1*, and *ARRB1*^18^. Tobacco use was associated with increased mutational burden, and head and neck SCC mutation rates were comparable to other smoking-related malignancies such as lung adenocarcinoma and small cell lung cancer^3^.

The differences in gene expression and DNA methylation features and discrepancies in methylation vs. expression trends may be due to several reasons. Chief among these are differences in the biological impact of methylation sites, differences in coverage of genes by epigenetic and transcriptome assays, and the impact of accounting for multiple testing. DNA methylation is only one of several regulatory mechanisms that influence gene expression and typically results in modest differences in gene expression that may have a cumulative impact over time. For example, while *GDNF* and *GPR15* expression data did not pass QC filtering, *GNG12* expression was successfully measured and displayed a modest and nominally significant inverse correlation with *GNG12* cg25189904 (r = −0.143, *p* = 0.027). This difference would not have been detected after correction for multiple testing and cg251189904 is unlikely to be the sole regulatory mechanism influencing GNG12 expression. In contrast, gene expression differences may represent the cumulative effects of a number of biological and environmental effects that may or may not include DNA methylation. Thus, it is not unexpected that the topmost differentially expressed genes would differ from the top most significantly differentially methylated positions.

The use of deep learning models to deconvolute histologic signatures is an emerging field in cancer biomarker development. Complex statistical modeling allows us to combine these histologic prediction scores with genetic biomarkers, as we have done in this study, to produce much more accurate biomarkers than ever before.

In this study our 16 gene targets, 3 epigenetic markers (*GPR15, GNG12, GDNF*) and 13 expression markers (*IGHA2, SCG5, RPL3L, NTRK1, CD96, BMP6, TFPI2, EFEMP2, RYR3, DMTN, GPD2, BAALC*, and *FMO3*), combined with histological modeling and clinical covariates, were 94% accurate in predicting the risk of 5-year mortality. This preliminary risk score was developed using two separate cohorts. Further validation is required in a prospective clinical cohort.

## Methods

### Patient selection and data collection

Institutional Review Board approval was obtained to create the de-identified patient databases at each respective institution (Loma Linda University, New York University, and University of Chicago). The study complied with all relevant ethical regulations including the Declaration of Helsinki. Informed consent was obtained from patients in the study. Enrollments were limited to only oral cavity sub-sites, including oral tongue, maxillary and mandibular gingiva, hard palate, floor of mouth, buccal mucosa, and lip mucosa. Clinical and pathologic stages were recorded based on the American Joint Committee on Cancer (AJCC) Eighth Edition Staging Manual^48^. All patients had biopsy-confirmed OSCC. De-identified patient demographic and clinical characteristics were used in the data interpretation. We collected the following information: age, sex, race, smoking (pack years) and alcohol use, staging, tumor location, pathologic characteristics [i.e., perineural invasion (PNI), lymphovascular invasion (LVI), margin status, histologic grade], and treatment modalities received in addition to surgery (i.e., neck lymphadenectomy, radiation therapy with or without chemotherapy).

### Illumina 450K methylation array analysis in TCGA and internal cohorts

We performed an analysis of methylation data from OSCC patients in the TCGA database. By design, TCGA generated data on genomic DNA and RNA from tumor sections. DNA methylation data pre-processing, quality control filtering, and normalization (inclusive of batch correction and surrogate variable analysis) were conducted employing the *minfi* package in R. Differential methylation analysis was performed using the *limma* package in R. The Illumina Infinium Methylation 450K Array data analyses are outlined in the workflow in Fig. 2A. Briefly, there were 225 samples with 485,512 probes. Probes that hybridized to the X or Y chromosomes were removed, leaving 473,864 probes. Additional probes that did not have *p*-value = 10^–8^ in at least 50% of the samples, or those that related to single nucleotide polymorphisms (SNPs), were removed. Only probes that determined methylation sites on a gene were retained, leaving 193,018 probes. *Limma* analysis was performed for this final set of probes.

Using the patient’s smoking status, we divided the patients into those who were never smokers, <10 pack years, or ≥10 pack years. The 10 pack year cut off is used in clinical trials to group patients into a high risk category^2^. Only 14 patients using tobacco belonged in the <10 pack years group, and therefore, to optimize the likelihood of identifying genome wide biomarkers with heavy tobacco use, we compared only the never smokers and the ≥10 pack years group. Using smoking status as a variable, we performed batch correction using surrogate variable analysis. Surrogate variables with a correlation of higher than 0.2 with survival status were excluded. Differentially methylated CpG for smoking status showing an adjusted p-value of <0.05 were considered for inclusion in the molecular component of the prognostic panel. To evaluate for enrichment of differentially methylated genes among pathways, pathway analysis was conducted using two complementary and overlapping annotations: gene ontology (GO^49^), Kyoto Encyclopedia of Genes and Genomes (KEGG^50^), and Reactome^51^. Pathway analysis was performed using *limma*, with significant (i.e., unadjusted *p*-value > 0.05) differentially methylated genes included in the analysis and non-significant genes specified as the “background universe”. Significantly perturbed pathways were declared at Bonferroni *p*-value < 0.05. For GO annotations, pathways were categorized further into biological process, molecular function, and cellular compartment. Differentially methylated pathways were evaluated by two visualizations of functional enrichment (i.e., dot plot and gene-concept networks) using the enrichplot package in R.

The 450 K methylation array data from the internal cohort (*n* = 40) was carried through the same pipeline to the methods described above.

### TCGA RNA sequencing and Internal Cohort Illumina HT12 gene expression array analyses

We determined differential gene expression based on RNA sequencing (RNASeq) data between never smokers and patients with ≥10 pack years (Fig. 2B). Raw gene counts were obtained from TCGA. Only genes with at least 10 counts in at least 90% of the samples were retained for analysis, totaling 15,234 genes. The Ensembl identifiers (ID) of the gene counts were annotated to Entrez IDs using the EnrichmentBrowser v 2.18.2 Package in R^52^, with 14,283 genes having an Entrez ID. Annotations for the genes were given using the Homo.sapiens v.1.3.1 package^53^. Correlation of RNASeq to CpG site methylation was performed using STATA. Functional pathway analyses were performed on the differentially expressed genes using GO, KEGG, and Reactome databases as described above.

The samples from the internal cohort underwent gene expression analysis using the Illumina HT-12 Gene Expression Array. Quality control filtering of array data was performed using ArrayQualityMetrics with any sample meeting any of three outlier detection methods removed from downstream analysis. *Oligo*^54^ was utilized for background correction, quantile normalization, and log_2_ transformation. Probes with a detection *p*-value > 0.05 removed. Correction for batch effects was performed using the Leek surrogate variable analysis method with the Bioconductor package *sva*^55^. Probes that did not map to a known gene were removed. Surrogate variable estimation was performed using control probes; control probes were then excluded before differential gene expression analysis of the remaining probes was performed. The Bioconductor package *limma*^56^, which fits a linear model, was employed for analysis of differential genes expression.

### Histology image processing

Scanned whole slide images (WSI) of hematoxylin and eosin-stained (H&E) tissue were acquired in SVS format from TCGA, followed by processing into individual tiles using the Slideflow (version 1.2.5) software package^57^. To process these large 1–3GB image files for input into the deep learning model, images are sectioned into hundreds of smaller images, or tiles. To enrich the deep learning model’s focus on tumor tissue rather than normal tissue, we only extract image tiles from regions of the WSI that a pathologist has annotated as tumor tissue. Areas of pathologist-annotated tumor are considered regions of interest (ROIs) within each WSI. When using H&E images from multiple institutions, we must take into consideration differences in the degree of H&E staining that occur due to variation in staining procedures across institutions. These staining differences are detectable by deep learning models and may bias results. When the model detects systematic differences in H&E stain, it may begin making predictions based on the prevalence of a disease state at the image’s originating institution via H&E stain proxy rather than capture biologically relevant histologic features. To overcome this limitation, we performed digital stain normalization using a modified Reinhard method, with brightness standardization disabled for improved computational efficiency^58^.

### Deep learning models

Deep learning models used an Xception-based architecture with ImageNet pretrained weights and three hidden layers of width 1024, with dropout of 0.1 after each hidden layer. Tiles received data augmentation with flipping, rotating, JPEG compression, and blur. Models were trained with Slideflow using the Tensorflow backend. To account for differences in the distribution of outcomes across contributing TCGA sites, we excluded images from sites that had only one outcome (Supplementary Table 1) and trained each model with 3-fold preserved-site cross-validation^59^. Hyperparameters were chosen based on the results of a limited hyperparameter sweep and previously reported model hyperparameters^57,59^. Models were trained over 5 epochs of data, using the Adam optimizer, with a learning rate of 10^–4^, a batch size of 16, sparse categorical cross-entropy loss, and no L2 regularization. All hyperparameters are listed in Supplementary Table 2.

### Derivation of patient level histology scores

After model training with 3-fold preserved-site cross-validation, we selected the best performing model across epochs from each fold. From the validation cohort in each fold we then extracted the pre-logit features from the second to last layer of the neural network. The pre-logit features act as a score representing the model’s confidence in a given WSI image’s association with a particular outcome. Multivariate analysis with additional data modalities integrated each patient’s pre-logit feature score as a measure that accounts for the deep learning model’s ability to determine the outcome of interest from H&E histology images.

### Statistical analyses

Statistical analyses were performed in STATA. Univariate analyses were performed to determine distributional characteristics and assess for randomness of the missing data (variables to be included in the final prognostic panel risk factor score had less than 5% missing values so imputation was not performed). Bivariate analyses with the primary outcome (vital status at 5 year follow-up) were performed on candidate variables, including smoking status, age and sex, with the outcome variable. For continuous variables (i.e., age), cut-offs were derived using the chi-square interaction detected by manual adjustment to ensure that cut-offs made sense clinically. Recursive partitioning was used to derive a final scoring system to predict survival status at 5-year follow-up with the goal of minimizing the number of misclassified values in the final cell while maximizing the simplicity of the score. Odds ratios at each decision node were rounded to the nearest integer to create the score. Operating characteristics of the derived risk score were calculated. The concordance statistic (c-index), equivalent to the area under the receiver operating curve (AUROC), was used to assess model discrimination and fit using the derived risk factor score to predict OSCC patients at risk for early mortality and morbidity^60^. The range of the c-index is from 0.5 (random concordance) to 1 (perfect concordance). While the initial derivation included all OSCC patients followed-up for 5 years, sensitivity analyses were performed to assess for bias from more high risk patients with an analysis that censored patients at 3-year follow-up.

Methylation analysis was performed according to a methylation state transition matrix^61^. A β-value of <0.3 indicated an unmethylated state, 0.33–0.75 a hemi-methylated state and >0.75 a fully methylated state. A gene was considered to be hypermethylated if the methylation level moved from a less methylated state to a more methylated state. Conversely, a gene was considered hypomethylated if there was a state change to a lower level. A change in methylation that did not have a state change was not considered significant^61^.

### Reporting summary

Further information on research design is available in the Nature Research Reporting Summary linked to this article.

### Supplementary information

Reporting Summary

Supplemental Material

## Data Availability

The study uses the publicly available data set from The Cancer Genome Atlas (TCGA) and an internal cohort. Researchers interested in accessing the data should contact the corresponding author. Restrictions apply to the availability of the internal Loma Linda University dataset, but all requests will be promptly evaluated based on institutional and departmental policies to determine whether the data requested are subject to intellectual property or patient privacy obligations. The Loma Linda University dataset can only be shared for non-commercial academic purposes and will require a data user agreement.

## Code availability

All code and models are made publicly available with an interactive user interface for class blending and latent space navigation at https://github.com/jamesdolezal/synthetic-histology, which includes all code necessary for reproducing results of this manuscript. The user interface provided is the same interface used during the educational teaching session. This code utilizes the software package Slideflow, available at https://github.com/jamesdolezal/slideflow.

## References

[1] Gulland, A., Oral cancer rates rise by two thirds. *BMJ* **355**, i6369 (2016). 10.1136/bmj.i6369 27887000

[2] Ferris, R. L., et al. Phase II Randomized Trial of Transoral Surgery and Low-Dose Intensity Modulated Radiation Therapy in Resectable p16+ Locally Advanced Oropharynx Cancer: An ECOG-ACRIN Cancer Research Group Trial (E3311). *J. Clin. Oncol*. **40**, 138–149 (2022). 10.1200/JCO.21.01752 PMC8718241 34699271

[3] Stransky N (2011). The mutational landscape of head and neck squamous cell carcinoma. Science.

[4] Poage GM (2011). Global hypomethylation identifies Loci targeted for hypermethylation in head and neck cancer. Clin. Cancer Res..

[5] Viet CT, Jordan RC, Schmidt BL (2007). DNA promoter hypermethylation in saliva for the early diagnosis of oral cancer. J. Calif. Dent. Assoc..

[6] Viet CT, Schmidt BL (2008). Methylation array analysis of preoperative and postoperative saliva DNA in oral cancer patients. Cancer Epidemiol. Biomark. Prev..

[7] Guerrero-Preston R (2014). Key tumor suppressor genes inactivated by “greater promoter” methylation and somatic mutations in head and neck cancer. Epigenetics.

[8] Ha PK, Califano JA (2006). Promoter methylation and inactivation of tumour-suppressor genes in oral squamous-cell carcinoma. Lancet Oncol..

[9] Huang MJ (2002). The correlation between CpG methylation and protein expression of P16 in oral squamous cell carcinomas. Int J. Mol. Med..

[10] Shaw RJ (2007). Quantitative methylation analysis of resection margins and lymph nodes in oral squamous cell carcinoma. Br. J. Oral. Maxillofac. Surg..

[11] Shaw RJ (2006). Promoter methylation of P16, RARbeta, E-cadherin, cyclin A1 and cytoglobin in oral cancer: quantitative evaluation using pyrosequencing. Br. J. Cancer.

[12] Smiraglia DJ (2003). Differential targets of CpG island hypermethylation in primary and metastatic head and neck squamous cell carcinoma (HNSCC). J. Med. Genet..

[13] Yeh KT (2002). The correlation between CpG methylation on promoter and protein expression of E-cadherin in oral squamous cell carcinoma. Anticancer Res..

[14] Li YF (2015). DNA methylation profiles and biomarkers of oral squamous cell carcinoma. Epigenetics.

[15] Viet CT (2021). The REASON Score: An Epigenetic and Clinicopathologic Score to Predict Risk of Poor Survival in Patients with Early Stage Oral Squamous Cell Carcinoma. Biomark. Res..

[16] Lin C (2017). GDNF secreted by nerves enhances PD-L1 expression via JAK2-STAT1 signaling activation in HNSCC. Oncoimmunology.

[17] Cao H (2020). The role of Glial cell derived neurotrophic factor in head and neck cancer. PLoS One.

[18] Chen Z (2020). From tobacco smoking to cancer mutational signature: a mediation analysis strategy to explore the role of epigenetic changes. BMC Cancer.

[19] Yang W (2021). Prognostic biomarkers and therapeutic targets in oral squamous cell carcinoma: a study based on cross-database analysis. Hereditas.

[20] Kolokythas A, Cox DP, Dekker N, Schmidt BL (2010). Nerve Growth Factor and Tyrosine Kinase A Receptor in Oral Squamous Cell Carcinoma: Is There an Association With Perineural Invasion?. J. Oral. Maxillofac. Surg..

[21] Weber M (2022). Beyond PD-L1-Identification of Further Potential Therapeutic Targets in Oral Cancer. Cancers.

[22] Kejner AE, Burch MB, Sweeny L, Rosenthal EL (2013). Bone morphogenetic protein 6 expression in oral cavity squamous cell cancer is associated with bone invasion. Laryngoscope.

[23] Kim SY (2019). Aberrantly hypermethylated tumor suppressor genes were identified in oral squamous cell carcinoma (OSCC). Clin. Epigenetics.

[24] Xu Y (2021). A ceRNA-associated risk model predicts the poor prognosis for head and neck squamous cell carcinoma patients. Sci. Rep..

[25] Roepman P (2005). An expression profile for diagnosis of lymph node metastases from primary head and neck squamous cell carcinomas. Nat. Genet.

[26] Yoon AJ (2020). MicroRNA-based risk scoring system to identify early-stage oral squamous cell carcinoma patients at high-risk for cancer-specific mortality. Head. Neck.

[27] van 't Veer LJ (2002). Gene expression profiling predicts clinical outcome of breast cancer. Nature.

[28] Fan C (2006). Concordance among gene-expression-based predictors for breast cancer. N. Engl. J. Med..

[29] Zeilinger S (2013). Tobacco smoking leads to extensive genome-wide changes in DNA methylation. PLoS One.

[30] Hidalgo M, Rowinsky EK (2000). The rapamycin-sensitive signal transduction pathway as a target for cancer therapy. Oncogene.

[31] Li J (2020). GNG12 regulates PD-L1 expression by activating NF-kappaB signaling in pancreatic ductal adenocarcinoma. FEBS Open Bio.

[32] Li L (2022). GNG12 Targeted by miR-876-5p Contributes to Glioma Progression Through the Activation of the PI3K/AKT Signaling Pathway. J. Mol. Neurosci..

[33] Yuan J (2021). Low GNG12 Expression Predicts Adverse Outcomes: A Potential Therapeutic Target for Osteosarcoma. Front. Immunol..

[34] Wiklund P (2019). DNA methylation links prenatal smoking exposure to later life health outcomes in offspring. Clin. Epigenetics.

[35] Gao X, Thomsen H, Zhang Y, Breitling LP, Brenner H (2017). The impact of methylation quantitative trait loci (mQTLs) on active smoking-related DNA methylation changes. Clin. Epigenetics.

[36] Adamczyk A (2021). GPR15 Facilitates Recruitment of Regulatory T Cells to Promote Colorectal Cancer. Cancer Res..

[37] Wu LH (2023). Construction and validation of a prognosis signature based on the immune microenvironment in gastric cancer. Front. Surg..

[38] Ohmomo H (2022). DNA Methylation Abnormalities and Altered Whole Transcriptome Profiles after Switching from Combustible Tobacco Smoking to Heated Tobacco Products. Cancer Epidemiol. Biomark. Prev..

[39] Huang BZ (2024). Epigenome-wide association study of total nicotine equivalents in multiethnic current smokers from three prospective cohorts. Am. J. Hum. Genet.

[40] Gao X, Jia M, Zhang Y, Breitling LP, Brenner H (2015). DNA methylation changes of whole blood cells in response to active smoking exposure in adults: a systematic review of DNA methylation studies. Clin. Epigenetics.

[41] Dogan MV (2015). Ethnicity and Smoking-Associated DNA Methylation Changes at HIV Co-Receptor GPR15. Front. Psychiatry.

[42] Huang Y (2021). Glial cell line-derived neurotrophic factor increases matrix metallopeptidase 9 and 14 expression in microglia and promotes microglia-mediated glioma progression. J. Neurosci. Res..

[43] Brown RW (2018). An analysis of the rewarding and aversive associative properties of nicotine in the neonatal quinpirole model: Effects on glial cell line-derived neurotrophic factor (GDNF). Schizophr. Res..

[44] Kotyuk E (2016). Association between smoking behaviour and genetic variants of glial cell line-derived neurotrophic factor. J. Genet.

[45] Ghantous Y, Schussel JL, Brait M (2018). Tobacco and alcohol-induced epigenetic changes in oral carcinoma. Curr. Opin. Oncol..

[46] Guerrero-Preston R (2009). Global DNA methylation: a common early event in oral cancer cases with exposure to environmental carcinogens or viral agents. P. R. Health Sci. J..

[47] Supic G, Kozomara R, Brankovic-Magic M, Jovic N, Magic Z (2009). Gene hypermethylation in tumor tissue of advanced oral squamous cell carcinoma patients. Oral. Oncol..

[48] Lydiatt WM (2017). Head and Neck cancers-major changes in the American Joint Committee on cancer eighth edition cancer staging manual. CA Cancer J. Clin..

[49] Ashburner M (2000). Gene ontology: tool for the unification of biology. The Gene Ontology Consortium. Nat. Genet..

[50] Kanehisa M (2002). The KEGG database. Novartis Found. Symp..

[51] Gillespie M (2022). The reactome pathway knowledgebase 2022. Nucleic Acids Res..

[52] Geistlinger L, Csaba G, Zimmer R (2016). Bioconductor’s EnrichmentBrowser: seamless navigation through combined results of set- & network-based enrichment analysis. BMC Bioinforma..

[53] TeamBC. *R Package: Homo.sapiens: Annotation package for the Homo.sapiens object* (TeamBC, 2015).

[54] Carvalho BS, Irizarry RA (2010). A framework for oligonucleotide microarray preprocessing. Bioinformatics.

[55] Leek JT, Johnson WE, Parker HS, Jaffe AE, Storey JD (2012). The sva package for removing batch effects and other unwanted variation in high-throughput experiments. Bioinformatics.

[56] Ritchie ME (2015). limma powers differential expression analyses for RNA-sequencing and microarray studies. Nucleic Acids Res.

[57] Dolezal JM (2021). Deep learning prediction of BRAF-RAS gene expression signature identifies noninvasive follicular thyroid neoplasms with papillary-like nuclear features. Mod. Pathol..

[58] Reinhard E, Adhikhmin M, Gooch B, Shirley P (2001). Color transfer between images. IEEE Computer Graph. Appl..

[59] Howard FM (2021). The impact of site-specific digital histology signatures on deep learning model accuracy and bias. Nat. Commun..

[60] Pencina MJ, D’Agostino RB (2015). Evaluating Discrimination of Risk Prediction Models: The C Statistic. JAMA.

[61] Hogan LE (2011). Integrated genomic analysis of relapsed childhood acute lymphoblastic leukemia reveals therapeutic strategies. Blood.

