# Supplementary material for: Artificial intelligence-based epigenomic, transcriptomic and histologic signatures of tobacco use in oral squamous cell carcinoma
Source: NPJ Precis Oncol. 2024 Jun 8;8:130. doi: 10.1038/s41698-024-00605-x (PMC11162452; doi:10.1038/s41698-024-00605-x)
Supplement: Supplementary file 1 — Reporting Summary [file 41698_2024_605_MOESM1_ESM.pdf]

nature portfolio

Corresponding author(s): Chi T. Viet NPJPRECISIONONCOLOGY-04409R

Last updated by author(s): Mar 8, 2024

Reporting Summary

Nature Portfolio wishes to improve the reproducibility of the work that we publish. This form provides structure for consistency and transparency in reporting. For further information on Nature Portfolio policies, see our Editorial Policies and the Editorial Policy Checklist.

Statistics

For all statistical analyses, confirm that the following items are present in the figure legend, table legend, main text, or Methods section.

n/a	Confirmed	
☐	☒	The exact sample size (*n*) for each experimental group/condition, given as a discrete number and unit of measurement
☐	☒	A statement on whether measurements were taken from distinct samples or whether the same sample was measured repeatedly
☐	☒	The statistical test(s) used AND whether they are one- or two-sided<br>*Only common tests should be described solely by name; describe more complex techniques in the Methods section.*
☐	☒	A description of all covariates tested
☐	☒	A description of any assumptions or corrections, such as tests of normality and adjustment for multiple comparisons
☐	☒	A full description of the statistical parameters including central tendency (e.g. means) or other basic estimates (e.g. regression coefficient) AND variation (e.g. standard deviation) or associated estimates of uncertainty (e.g. confidence intervals)
☐	☒	For null hypothesis testing, the test statistic (e.g. *F*, *t*, *r*) with confidence intervals, effect sizes, degrees of freedom and *P* value noted<br>*Give P values as exact values whenever suitable.*
☐	☒	For Bayesian analysis, information on the choice of priors and Markov chain Monte Carlo settings
☐	☒	For hierarchical and complex designs, identification of the appropriate level for tests and full reporting of outcomes
☐	☒	Estimates of effect sizes (e.g. Cohen's *d*, Pearson's *r*), indicating how they were calculated

Our web collection on statistics for biologists contains articles on many of the points above.

Software and code

Policy information about availability of computer code

Data collection	Data collection and analysis software are included int the Methods section, organized by each omic approach (epigenomic, gene expression or digital pathomics).
Data analysis	see above

For manuscripts utilizing custom algorithms or software that are central to the research but not yet described in published literature, software must be made available to editors and reviewers. We strongly encourage code deposition in a community repository (e.g. GitHub). See the Nature Portfolio guidelines for submitting code & software for further information.

Data

Policy information about availability of data

All manuscripts must include a data availability statement. This statement should provide the following information, where applicable:

- Accession codes, unique identifiers, or web links for publicly available datasets
- A description of any restrictions on data availability
- For clinical datasets or third party data, please ensure that the statement adheres to our policy

Due notice has been made regarding the policies and recommendations on data and resource sharing. Intellectual property and genomic data generated through the project will be administered in accordance to these policies and the policies of the Loma Linda University Office of Technology Transfer. This office provides guidance on issues and procedures related to technology transfer, and will be responsible for managing all requests for resources and intellectual property

generated by the university. It is planned that results from this project will be presented at national research conferences, and will also be drafted in manuscript form and submitted for publication during the R01 funding period. Generated data will be shared publicly, and accepted manuscripts will be made available on PubMed Central as mandated by the NIH public access policy. The generated data will be submitted to the NCI Genomic Data Commons as appropriate. Research data from participants will be redacted to remove all potential identifiers before data sharing.

Research involving human participants, their data, or biological material

Policy information about studies with human participants or human data. See also policy information about sex, gender (identity/presentation), and sexual orientation and race, ethnicity and racism.

Reporting on sex and gender	Sex and gender were reported in the manuscript.
Reporting on race, ethnicity, or other socially relevant groupings	Race, ethnicity, socioeconomic status, education level, annual income were all collected from prospectively enrolled patients. Race/ethnicity were reported in this manuscript.
Population characteristics	All patients had biopsy proven oral squamous cell carcinoma. Please see manuscript for the mean age of each subgroup.
Recruitment	Patients were recruited from the University Tumor Board list.
Ethics oversight	Loma Linda University

Note that full information on the approval of the study protocol must also be provided in the manuscript.

Field-specific reporting

Please select the one below that is the best fit for your research. If you are not sure, read the appropriate sections before making your selection.

☒ Life sciences ☐ Behavioural & social sciences ☐ Ecological, evolutionary & environmental sciences

For a reference copy of the document with all sections, see nature.com/documents/nr-reporting-summary-flat.pdf

Life sciences study design

All studies must disclose on these points even when the disclosure is negative.

Sample size	Sample size for the publicly available TCGA data set was determined by identifying all available oral cavity squamous cell carcinoma cases. Sample size for the internal cohort was determined by using every patient that was enrolled to optimize the ability to find significant biomarkers.
Data exclusions	No data was excluded.
Replication	Data integrity and replication were described in Methods.
Randomization	This is not relevant.
Blinding	This is not relevant.

Reporting for specific materials, systems and methods

We require information from authors about some types of materials, experimental systems and methods used in many studies. Here, indicate whether each material, system or method listed is relevant to your study. If you are not sure if a list item applies to your research, read the appropriate section before selecting a response.

Materials & experimental systems

n/a	Involved in the study
☒	☐ Antibodies
☒	☐ Eukaryotic cell lines
☒	☐ Palaeontology and archaeology
☒	☐ Animals and other organisms
☒	☐ Clinical data
☒	☐ Dual use research of concern
☒	☐ Plants

Methods

n/a	Involved in the study
☒	☐ ChIP-seq
☒	☐ Flow cytometry
☒	☐ MRI-based neuroimaging

Plants

Seed stocks	n/a
Novel plant genotypes	n/a
Authentication	n/a
