# Supplementary material for: Artificial intelligence-based epigenomic, transcriptomic and histologic signatures of tobacco use in oral squamous cell carcinoma
Source: NPJ Precis Oncol. 2024 Jun 8;8:130. doi: 10.1038/s41698-024-00605-x (PMC11162452; doi:10.1038/s41698-024-00605-x)
Supplement: Supplementary file 2 — Supplemental Material [file 41698_2024_605_MOESM2_ESM.docx]

**Supplementary Table 1**

| **TCGA Donating Site** | **Number of Non-smoker Images** | **Number of Smoker Images** |
| --- | --- | --- |
| BA | 4 | 12 |
| BB | 5 | 1 |
| C9* | 2 | 0 |
| CN | 7 | 20 |
| CQ | 11 | 22 |
| CV | 23 | 26 |
| CX* | 0 | 4 |
| D6 | 2 | 7 |
| DQ | 1 | 4 |
| F7 | 1 | 5 |
| H7 | 0 | 2 |
| HD | 4 | 4 |
| HL* | 0 | 1 |
| IQ | 2 | 6 |
| KU | 1 | 1 |
| MT | 3 | 2 |
| P3 | 5 | 5 |
| QK | 3 | 4 |
| RS* | 1 | 0 |
| T2 | 1 | 2 |
| T3 | 0 | 1 |
| UF | 3 | 4 |
| UP* | 1 | 0 |
| WA* | 0 | 2 |

**Supplementary Table 1** shows the site, or institution, that donated the images to TCGA as identified by the two-alphanumeric code. Next to each site, we list the number of histology available images from that site belonging to either a smoker or non-smoker. Models were trained using 3-fold cross validation with images from the TCGA. There was not an additional external validation cohort used to develop the histology model.

* WSI from these sites were excluded from analysis due to bias introduced by class imbalance. By removing these images, we limit the possibility that the model learns features that predict where the image was derived (such as age of the sample, the type of scanner used to capture the image, degree of H&E staining) as a proxy for smoking status.

**Supplementary Table 2**

| **Hyperparameter** |  |
| --- | --- |
| Augmentation | Flip, Rotate, JPEG Compression, Blur |
| Batch Size | 16 |
| Dropout | 0.1 |
| Early Stopping | Yes |
| Early Stopping Method | Accuracy |
| Early Stop Patience | 0 |
| Epochs | 5 |
| Hidden Layer Width | 1024 |
| Hidden Layers | 3 |
| L1 | 0 |
| L1 Dense | 0 |
| L2 | 0 |
| L2 Dense | 0 |
| Learning Rate | 0.0001 |
| Learning Rate Decay | 0.97 |
| Learning Rate Decay Steps | 100000 |
| Loss | Sparse Categorical Cross Entropy |
| Normalizer | Reinhard (modified) |
| Optimizer | Adam |
| Pooling | Average |

**Supplementary Table 3**

| **Outcome** | **Level** | **k-fold** | **AUROC** | **AUPRC** | **PPV** | **NPV** | **Sensitivity** | **Specificity** | **Epoch** |
| --- | --- | --- | --- | --- | --- | --- | --- | --- | --- |
| 3-Fold CV  Smoking Status | Patient | 1 | 0.62 | 0.71 | 0.69 | 0.74 | 0.74 | 0.41 | 5 |
|  |  | 2 | 0.49 | 0.65 | 0.65 | 0.65 | 0.60 | 0.45 | 4 |
|  |  | 3 | 0.52 | 0.65 | 0.66 | 0.67 | 0.60 | 0.52 | 3 |
|  | Tile | 1 | 0.45 | 0.64 | 0.52 | 0.52 | 0.42 | 0.53 | 5 |
|  |  | 2 | 0.51 | 0.53 | 0.63 | 0.63 | 0.60 | 0.43 | 4 |
|  |  | 3 | 0.52 | 0.61 | 0.70 | 0.71 | 0.61 | 0.42 | 3 |
| 3-Fold CV  Vital Status | Patient | 1 | 0.53 | 0.27 | 0.66 | 0.69 | 0.65 | 0.51 | 5 |
|  |  | 2 | 0.48 | 0.45 | 0.61 | 0.61 | 0.50 | 0.57 | 5 |
|  |  | 3 | 0.54 | 0.27 | 0.71 | 0.73 | 0.64 | 0.43 | 4 |
|  | Tile | 1 | 0.49 | 0.28 | 0.56 | 0.57 | 0.65 | 0.39 | 5 |
|  |  | 2 | 0.45 | 0.25 | 0.60 | 0.60 | 0.40 | 0.57 | 5 |
|  |  | 3 | 0.51 | 0.50 | 0.70 | 0.72 | 0.67 | 0.37 | 4 |
| No CV  Smoking Status | Patient | – | 0.67 | 0.80 | 0.66 | 0.70 | 0.71 | 0.39 | 1 |
|  | Tile | – | 0.68 | 0.61 | 0.63 | 0.66 | 0.67 | 0.40 | 1 |
| No CV Vital Status | Patient | – | 0.37 | 0.30 | 0.15 | 0.26 | 0.13 | 0.66 | 1 |
|  | Tile | – | 0.41 | 0.34 | 0.28 | 0.33 | 0.25 | 0.63 | 1 |

Summary of model performance. Pre-logit feature activation scores were extracted from each 3-fold CV with Site Preservation model and incorporated into multivariate models. UMAP plots and UMAP mosaic plots were generated with predictions from the Full Dataset Model without CV. Sensitivity and specificity were calculated from manual thresholding of AUROC plots.


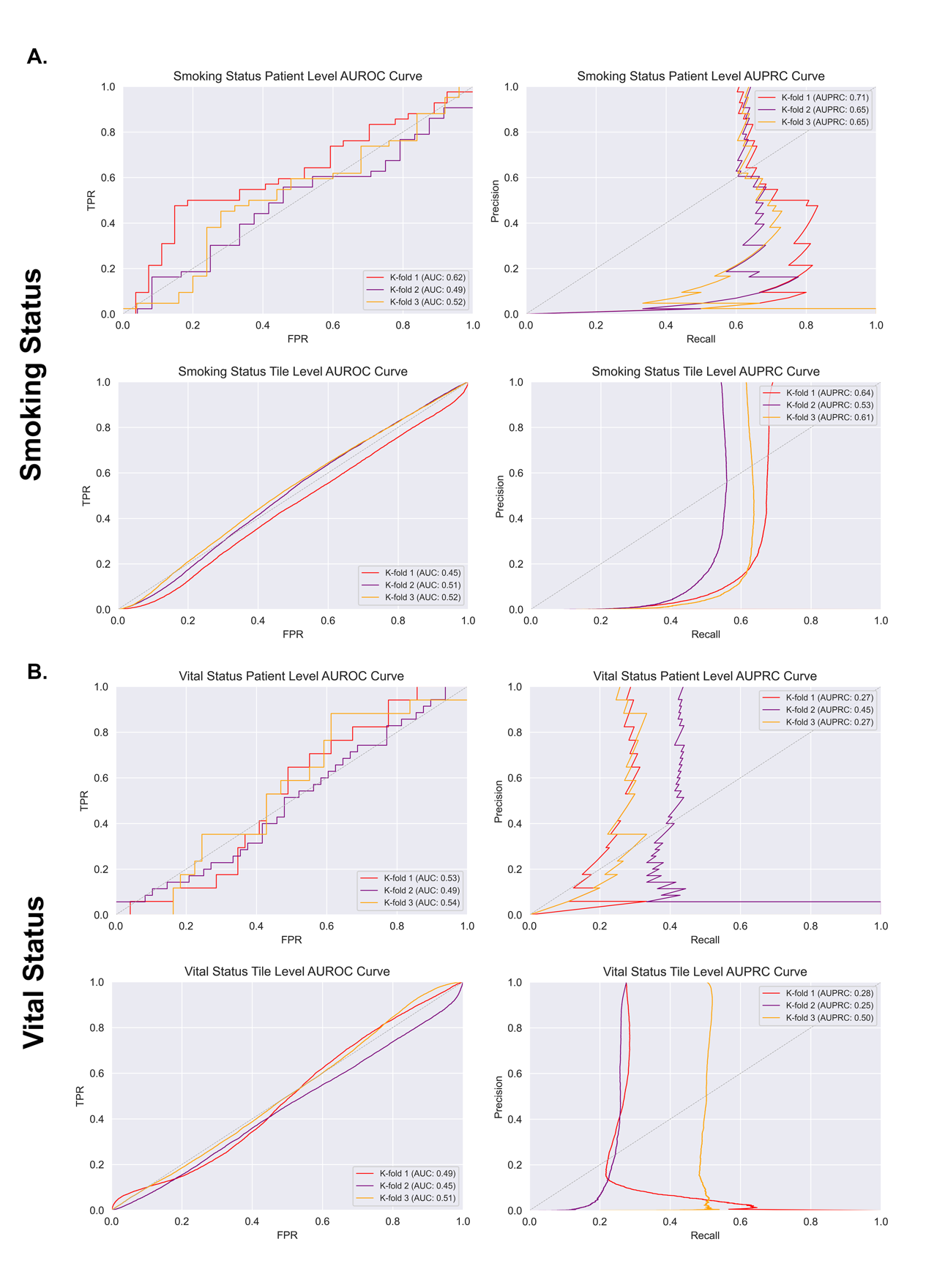


**Supplementary Figure 1.** Patient level and tile level receiver operating curves (ROC) and precision-recall curves (PRC) from each fold in the 3-fold CV with site preservation models with the outcomes of (A) smoking status and (B) vital status.


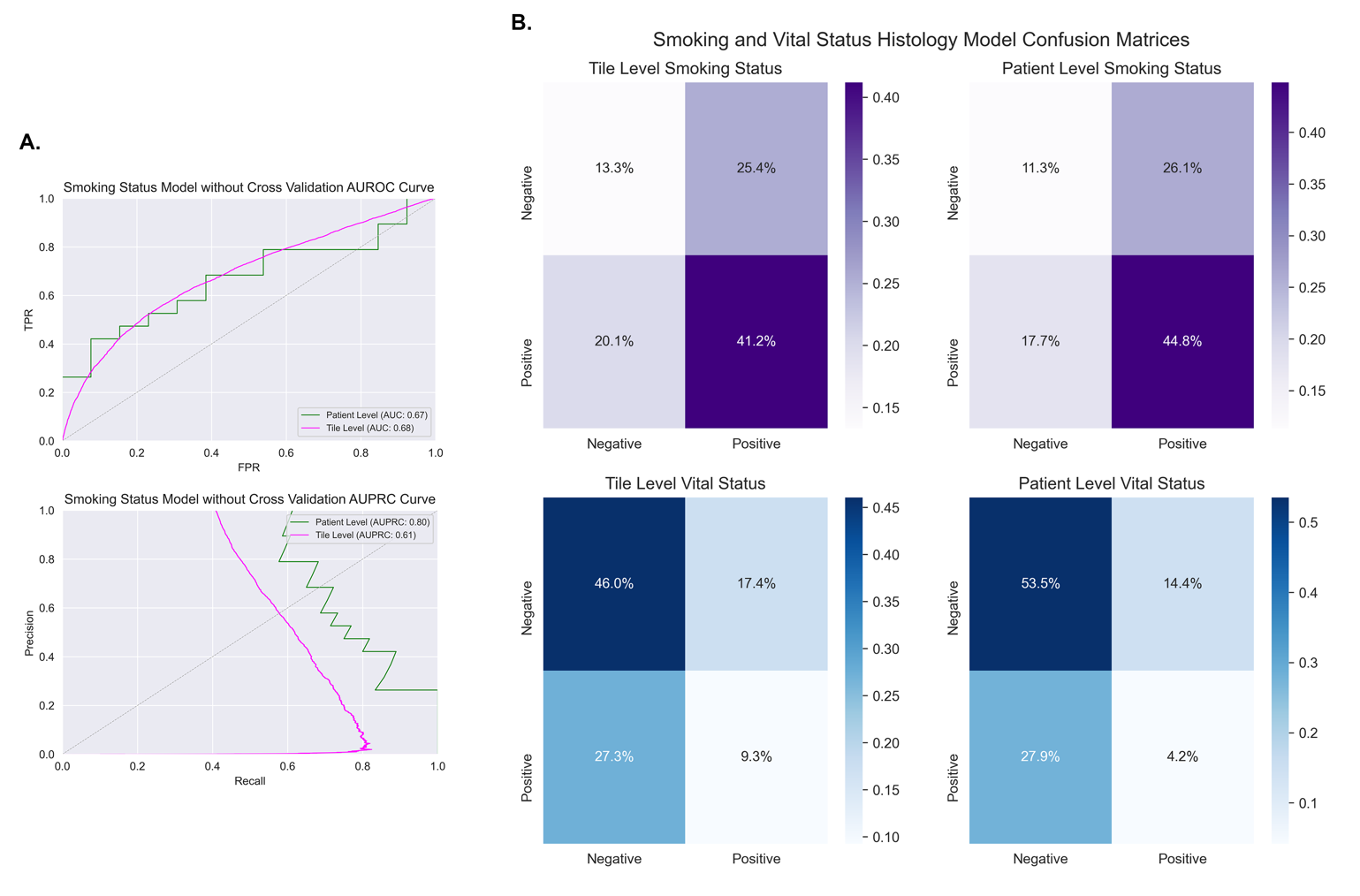


**Supplemental Figure 2**. (A) Patient level and tile level smoking status prediction ROC curves and precision-recall curves from the full dataset model without CV. (B) Confusion matrices for patient level and tile level smoking status predictions.
